# Diagnostic Management of Pancreatic Cancer

**DOI:** 10.3390/cancers3010494

**Published:** 2011-01-31

**Authors:** Emanuele Dabizzi, Mauricio Saab Assef, Massimo Raimondo

**Affiliations:** 1 Division of Gastroenterology and Hepatology, Mayo Clinic Florida, 4500 San Pablo Road, Jacksonville, Florida 32224, USA; E-Mail: emadabi@yahoo.it; 2 Faculdade de Ciências Médicas da Santa Casa de São Paulo, Rua Dr. Cesário Motta Jr. #61 Cep: 01221-020, São Paulo, Brazil; E-Mail: msassef@terra.com.br

**Keywords:** endosonography, pancreas, pancreatic carcinoma

## Abstract

Pancreatic cancer is one of the most deadly solid tumors, with an overall 5-year survival rate of less than 5%. Due to a non-specific clinical presentation, it is often diagnosed at an advanced stage and is rarely amenable for curative treatment. Therefore early diagnosis and appropriate staging are still essential to define the best care and to improve patient survival. Several imaging modalities are currently available for the evaluation of pancreatic cancer. This review focuses on different techniques and discusses the diagnostic management of patients with pancreatic cancer. This review was conducted utilizing Pubmed and was limited to papers published within the last 5 years. The search key words pancreatic cancer, pancreatic adenocarcinoma, pancreatic tumors, diagnosis, radiology, imaging, nuclear imaging, endoscopy, endoscopic ultrasound and biochemical markers were used.

## 1. Introduction

Pancreatic cancer is considered to have one of the worst prognoses of all solid malignancies. It has a relatively high incidence as well, making it one of the top ten incidence cancer in Europe and USA, with an overall 5-year survival rate of less than 5% [1,2].

Several studies have shown that patients with early stage pancreatic lesions (<3 cm) without lymphatic invasion have a significantly better prognosis with a 5-year survival rate of up to 25–30% following surgical resection. These results support that early tumor detection is still essential to improve patient survival [3,4].

The majority of pancreatic exocrine tumors are adenocarcinomas, arising from the ductal epithelium. These tumors have a non-specific clinical presentation, mainly depending on the location and the stage of the tumor.

In order to complete this review, we performed a Pubmed search and limited our results to papers published within the last 5 years. We also used the following key words: Pancreatic cancer, pancreatic adenocarcinoma, pancreatic tumors, diagnosis, radiology, imaging, nuclear imaging, endoscopy, endoscopic ultrasound and biochemical markers. We also referred to our expertise having a combined 50 years of experience among the three authors.

## 2. Epidemiology and Risk Factors

Pancreatic cancer represents the tenth most frequent cancer in Europe, and ranks eight on this list of cancer-related death. However in the United States, the estimated new cases in 2010 could be as high as 43,140 cases and 36,800 patients will die from the disease [2], making it the tenth most incident cancer and the fourth leading cause of cancer death. The age-adjusted incidence rate is slightly greater in men than in women [2,5]. The median age at diagnosis is 73 years, with the incidence increasing with age [6]. This disease is rare before the age of 40 years [5].

The causes underlying pancreatic cancer are still uncertain, but some risk factors have been identified. Tobacco smoking is the only element with strongly suspected causative role in pancreatic neoplasia. Smokers have up to 3.6% increased risk of pancreatic tumor [1]. Limited data points to other possible factors such as alcohol, diet high in cholesterol and fat, low serum folate levels, obesity, long-standing diabetes mellitus, chronic pancreatitis and blood type A, B or AB as compared with blood type O [1,5].

About 10% of patients with pancreatic cancer have a positive family history for this disease, which can be part of a well-defined cancer-predisposing genetic syndrome, as well as germ-line mutations [1].

## 3. Biology of Pancreatic Tumor

The main histology pattern of pancreatic cancer is infiltrating ductal adenocarcinoma, accounting for up to 90% of all pancreatic malignancies. The remaining 10% is represented by acinar cell carcinoma and pancreatoblastoma, mainly occurring in children [6].

Some other rare histologic patterns of pancreatic cancer that have been described are adenosquamous carcinoma, colloid carcinoma, hepatoid carcinoma, medullary carcinoma, signet-ring cell carcinoma, undifferentiated and undifferentiated with osteoclast-like cells carcinoma [5]. The infiltrating ductal adenocarcinoma originates in the ductal epithelium and evolves from low grade through high grade dysplastic lesions, up to invasive carcinoma, as a result of consecutive gene mutations.

Most of the pancreatic neoplasms carry one or more of four genetic aberrations. Up to 90% of carcinomas show an activating mutation in the k-*ras* oncogene, which inactivates the CDKN2A gene in 95% [1]. These different genetic mutations, combined in different ways lead to the formation of a desmoplastic reaction, due to the activation of myofibroblasts, which results in a dense stroma. These cells can regulate both the composition of extracellular matrix as well as the poor vascularization of neoplastic tissue, which is a peculiar characteristic of pancreatic cancer [1].

As in colorectal cancer, with the “adenoma-carcinoma” sequence, different histological pre-malignant lesions have been described [5]. These lesions when histologically characterized include pancreatic intraepithelial neoplasia (PanIN), intraductal papillary mucinous neoplasm (IPMN) and mucinous cystic neoplasm (MCN). These three most common pre-malignant lesions have been demonstrated as a multistep morphologic and genetic progression towards invasive pancreatic carcinoma, although each lesion is characterized by distinctive clinicopathological and genetic characteristics [4].

PanIN are microscopic lesions of the smaller pancreatic ducts (<5 mm). They can be papillary or flat and they are composed of columnar or cuboidal cells with varying amount of mucin [5,7]. They are genetically characterized by the same aberrations found in infiltrating adenocarcinoma [7].

IPMN are macroscopic mucin-producing epithelial lesions of the main pancreatic duct or its branches. They are frequently characterized by a papillary architecture [5].

MCN are macroscopic cystic precursors, which occur mostly in women with a peculiar ovarian type stroma [4].

Although just IPMN and MCN are macroscopically visible with current diagnostic imaging, early detection of these pre-neoplastic lesions is essential to reduce the high mortality rate of pancreatic cancer.

## 4. Clinical Presentation

The clinical presentation of pancreatic cancer can widely vary, due to tumor location and disease stage. Tumors developing in the head of the pancreas cause obstructive jaundice and weight loss, which can be profound and associated with diarrhea and steatorrhea. Abdominal discomfort and nausea are also common.

Tumors of the body and tail usually present with abdominal pain and weight loss. Pain is quite frequent in pancreatic cancer (up to 80–85%). It is reported as a dull ache, deep, coming from the upper abdomen, radiating through the back. Rarely, pancreatic tumors can cause duodenal obstruction or gastrointestinal bleeding [1,6]. Up to 80% of patients with pancreatic cancer have hyperglycemia or are diabetic. Other vague systemic signs and symptoms include asthenia, anorexia and rarely superficial venous thrombosis, panniculitis, liver-function abnormalities, gastric-outlet obstruction and increased abdominal girth.

Blood tests are generally non-specific and may include mild abnormalities of liver chemistry, hyperglycemia and anemia, with the exception of some tumor markers such as carbohydrate antigen 19-9 (CA 19-9) and carcino-embryonic antigen (CEA), useful more in diagnosing cancer recurrence than to screen patients [8].

## 5. Tumor Staging and Criteria for Resectability

The most used staging system for pancreatic cancer is the TNM, proposed by AJCC/UICC, showed in Table 1. Based on this, the patients can be stratified in groups, as shown in Table 2.

Surgical resection is the only potentially curative treatment for pancreatic adenocarcinoma, although most of the patients are found with unresectable disease at the time of surgery. It should be limited to those patients without metastatic disease and in which the entire lesion can be resected with negative margins. In some patients the only obstacle for a radical resection is represented by the involvement of the portal or superior mesenteric vein. In the past, this was considered a contraindication for pancreaticoduodenectomy. This was due to technical difficulties and poor long term survival rate [10]. Currently, venous resection and reconstruction can be performed with morbidity and mortality rates similar to surgery in patients without vascular invasion [10] that showed survival advantage.

Reflecting these management changes, isolated venous involvement is considered a locally invasive tumor, but potentially resectable (T3). However, the involvement of the celiac artery or superior mesenteric artery remains a T4 lesion, due to the concomitant presence of extensive celiac or mesenteric neural invasion. It is important to note that in carefully selected cases, early arterial invasion can be considered resectable [11].

## 6. Diagnosis

The differential diagnosis of exocrine pancreatic neoplasia should include focal chronic pancreatitis, autoimmune pancreatitis, and pancreatic endocrine tumors over other rare conditions and metastatic diseases (*i.e.,* renal cell carcinoma). Although different modalities are currently available, the differentiation between benign lesions and malignant neoplasms is still challenging.

## 7. Tumor Markers

A limited role is still assigned to tumor markers. This is due to their low sensitivity and specificity as their serum levels can help to assess prognosis and therapeutic efficacy in pancreatic cancer, suggesting cancer recurrence. CA 19-9 is a glycoprotein expressed on the surface of normal pancreatic and biliary ductal cells. Increased serum levels are found in patients with pancreatic cancer, but also with hepatocellular and ovarian carcinoma, bronchial, colon and gastric cancer as well as cholestatic disease, chronic pancreatitis and other inflammatory disorders [8]. Therefore, the sensitivity and specificity of CA 19-9 are quite variable, ranging from 67 to 92% and 68 to 92%, respectively. Some limitations of CA 19-9 sensitivity can be related to lesion size and genetic basis. Small cancers (<2 cm) are associated with serum elevation just in 50% of cases [8]. Besides, patients with negative Lewis blood group antigen are unable to synthesize this glycoprotein. Therefore, they have negative CA 19-9 serum levels because of genetic reasons [8].

CEA is expressed in normal mucosal cells. Elevated serum levels are also found in most of the gastrointestinal cancers such as colorectal and pancreatic cancers. However, CEA is over expressed in benign conditions like peptic ulcer, pancreatitis, biliary obstruction, inflammatory bowel disease and in cigarette smokers as well [8].

Some data suggest a role for the beta subunit of human chronic gonadotropin (beta-hcg) and CA 72-4 as prognostic markers [12]. Recently, molecular markers have been proposed to improve the diagnostic yield of conventional cytological samples, especially in order to distinguish benign form malignant lesions [13]. They include mutations of k-*ras* and an assessment of telomerase activity. Several studies showed a significant increment in the sensitivity and overall accuracy of conventional cytology, when k*-ras* mutations analysis is performed [13,14]. Detection of telomerase activity, usually absent in normal cells, can also increase the sensitivity of conventional cytology from 85 to 98%, maintaining the specificity of 100% [15].

## 8. Imaging

The main aim of imaging tests is early detection as well as an accurate staging of lesion extension and possible vessel invasion. This is done in order to choose the best clinical and therapeutic management. Multiple methods have been developed in the last few decades to improve pancreatic cancer detection. However, taken individually, these imaging tests have variable sensitivity.

## 9. Endoscopic Retrograde Cholangiopancreatography (ERCP)

This procedure allows for visualization of the hepatobiliary tree, sampling of pure pancreatic juice and assessment for genetic analysis of tissue from biopsies and brushings [8]. The role of ERCP in the diagnosis of pancreatic cancer is considerably reduced, if compared to the past. Due to the post-procedural risk of pancreatitis, it is mainly a therapeutic modality with stent placement in patients with obstructive disease, whereas its diagnostic role has been replaced by EUS and MRCP, where available [8].

## 10. Transabdominal Ultrasound US

Transabdominal US is commonly the first line imaging test for patients with suspected pancreatic cancer, due to its wide availability, safety and low cost. Limitations of pancreas visualization are represented by the patient's body habitus, overlying bowel gas, as well as sonographer experience [8]. The sensitivity of US in detecting pancreatic tumors can be up to 95%. If the lesion is more than 3 cm. US can examine biliary tract dilation and establish the level of obstruction [8].

The use of harmonic imaging and contrast medium can enhance the lesions characterization reducing artifacts. Recent studies have shown an increased sensitivity for small lesions (<2 cm), similar to CT scan [8,16], as well as a better characterization (adenocarcinoma and neuro-endocrine tumors), and vascular staging [17].

Though US is a sensitive method to detect small liver metastases [8], US alone can't guarantee enough accuracy in diagnosis and staging of pancreatic tumors. Therefore, it should be considered a useful tool for initial assessment in suspected pancreatic lesions.

## 11. Endoscopic Ultrasound (EUS)

Since its first advent in the 1980s, EUS appeared as a promising imaging method for the study of pancreatobiliary diseases. With several technological improvements, such as the introduction of electronic transducers and color-Doppler capability, EUS is able to gain accurate images, with high negative predictive value, ranging from 87 to 100% [18,19].

Due to the small distance between the probe and the pancreas and the capability of tissue acquisition, EUS is considered the best imaging technique for the study of pancreas [8]. This is especially true when other cross-sectional modalities render controversial results. EUS can assess tumor invasion and presence of metastatic lymph nodes with high accuracy. For better diagnostic yield, EUS should be performed after contrast enhanced CT and before ERCP [8,20].

Pancreatic cancers can have different ultrasound appearance, depending both on tumor location and on the lesion intrinsic nature (*i.e.,* cystic component). Pancreatic cancer is usually seen as ill-defined, hypoechoic mass. It can have inhomogeneous pattern and can cause dilation of the distal pancreatic duct [8]. Sensitivity of EUS in pancreatic mass detection can reach 97%, with a better yield than transabdominal US and conventional CT scan, mainly with small size lesions (<3 cm) [8]. Despite high sensitivity, specificity is quite limited, especially in the differential diagnosis with inflammatory processes, which can mimic the same sonographic morphology [8]. The reported accuracy of T staging ranges from 63% to 94%, whereas N staging varies between 41 and 86%, superior to both transabdominal US and CT [21].

EUS-guided Fine Needle Aspiration (EUS-FNA) is an additional tool for cytological tissue sampling of suspicious lesions and regional lymph nodes assessment. Its feasibility varies from 90% to 98%, whereas accuracy of collecting diagnostic specimens ranges from 80% to 95% [22]. Its sensitivity and specificity for solid pancreatic masses diagnosis are 95% and 92% respectively, with a positive predictive value of 98% and a negative predictive value of 80% and an accuracy of 94% [23]. However, the sensitivity and specificity rate can be surprisingly variable due to a difficult cytology interpretation of low cellular samples, because well differentiated tumors, extensive fibrosis (desmoplasia) and the presence of calcifications that can prevent an adequate tissue sampling and analysis [13]. Despite these limitations, EUS-FNA can still yield better sensitivity and specificity than ERCP brushing and CT/US-guided FNA in the diagnosis of pancreatic cancer [13,24]. Moreover, EUS-FNA can visualize and sample small lesions as neuroendocrine tumors or pancreatic metastasis, difficult to reach with a percutaneous access [13,25]. Overall diagnostic yield as well as sensitivity, specificity and accuracy, seem not to be influenced by tumor size [26].

When alternative techniques of tissue sampling are possible, EUS-FNA should be the preferred method for several reasons: it is the most cost-effective modality as primary approach and it is able to change patient management as previously reported [27,28]. Moreover, EUS-FNA has a lower risk of needle tract seeding when compared with a percutaneous approach due to a short needle track, which does not pass through peritoneal or pleural surfaces. Exceptions to this occur when sampling liver lesions and pancreatic body/tail lesions where the peritoneum is breached [22].

Despite these results, the indications for performing EUS-FNA in pancreatic cancer are still controversial. If widely accepted in patients with unresectable pancreatic tumors, some controversy may persist if preoperative tissue diagnosis is needed, as in the case of resectable disease [21].

EUS-FNA should be performed before consideration for chemo-radiotherapy in patients with distant metastasis and advanced locoregional disease. Tissue diagnosis in resectable patients should be considered in order to exclude other pathology (*i.e.,* lymphoma, small cell carcinoma, *etc.*) and to optimize surgical management (organ-preserve surgery) [13].

EUS-FNA is reported as an accurate and safe modality in suspected pancreatic cancer, with an acceptable risk. Few studies have assessed the overall risk rate. The main complication related to the procedure is pancreatitis, ranging from 0.5% to 2%, followed by abdominal pain, fever, infection, bleeding and perforation [21,29].

Eloubeidi *et al.* [29] prospectively documented a 2.4% overall rate of major complications, related to the procedure: acute pancreatitis (0.85%), abdominal pain (0.56%) and fever (0.85%). The authors also assessed the potential risk factors for FNA complications, but history of acute or chronic pancreatitis was not associated with higher risk. They evaluated the possible implication of the number of needle passes, which did not reach statistical significance [29].

To date, no studies have evaluated the role of needle size in causing complication. Previous trials conducted with a percutaneous technique showed that a higher needle size can be associated to a higher risk of complication [29]. The infection risk for puncturing solid pancreatic masses is minimal and antibiotic prophylaxis is not necessary [13].

EUS-guided core needle biopsy (EUS-CNB) is another tissue sampling method, developed to overcome the limitations of EUS-FNA, especially the low cellularity sample. It is a device with a 19 gauge needle and an 18 mm specimen tray, which can collect a tissue core for histologic assessment [30], useful also for gene and immuno-hystochemical analysis [13]. It may improve the diagnostic yield of EUS, especially in lymphomas, where a cytology specimen does not allow assessment on tissue architecture, as well as subepithelial tumors, autoimmune pancreatitis, tuberculous lymphadenopathy and microcystic pancreatic tumors [31].

Only few studies evaluated the role of EUS-CNB in diagnosis of pancreatic masses. This method, limited by device design and stiffness, seems feasible through a transgastric approach, whereas it is sometimes problematic and unsuccessful transduodenally. Punctures occur where more scope angulation is required [13,31].

Several studies have evaluated the diagnostic yield and accuracy of different size needles, both prospectively and retrospectively, with variable results. The needles available are 25 g, 22 g, 19 g and 19 g trucut. Sakamoto *et al.* prospectively compared three needles (25 g, 22 g, 19 g-Trucut) per patient, and evaluated the technical success and accuracy of these different sampling modalities [32]. The technical success rate of 25 g needle was significantly higher than the trucut (p < 0.05). This result was evident for uncinate process lesions, where the 25 g needle was useful in 100% of cases, while 19 g needle was ineffective in all cases and the 22 g was successful in 33%. Similar results were achieved for pancreatic head lesions, where no significant difference was observed for tail lesions [32]. Relevant data were also gained about the overall diagnostic accuracy for the three needles which was 92% for the 25 g, 80% for the 22 g and 54% for the 19 g. Slight divergences were reported by Lee *et al.,* who prospectively compared 25 g and 22 g. They observed no significant difference in cellularity between the two needles, although they agreed with others that success of FNA with larger needle is limited by the site of the lesion [33,34].

Overall, these data suggest that the choice of needle type in pancreatic masses should depend on the site of the lesion [32,33], arguing that thinner needles offer more advantages, with greater safety and flexibility for puncture than thicker ones. They however, could provide smaller specimens for diagnosis [32,33].

The number of passes is still a controversial issue: The ideal number is not defined, but it should guarantee enough cells/tissue for the final analysis. Retrospective studies reported a wide range (2–6) of needle required passes. Prospectively, LeBlanc *et al.* [35] suggested that 7 passes for pancreatic masses and 5 passes for lymph nodes are needed for cytological adequacy [35]. However, Pellise *et al.* [36] reported that the efficacy of EUS-FNA reaches a plateau on the forth pass after a progressive increment [36].

The onsite presence of a cytotechnologist can certainly help to obtain an adequate sample, especially for lymph nodes biopsies [37,38]. Specimens obtained should be in part air-dried and stained with Diff-Quick method for the onsite evaluation; while the other part should be fixed in 95% alcohol for Papanicolaou's staining. Any additional material can be collected in saline solution or formalin for cell block [39].

Elastography is a novel imaging technique, which can assess the tissue stiffness in real-time using conventional ultrasound with modified software instruments. Based on the different pathologic tissue structure and consequent distorsion when they are externally slightly compressed, this technique applies a pressure to a part of an organ, with the result of a different degree of deformation visualized as a scale of colors [40,41]. It was firstly used for the study of breast, prostate gland and liver. Recently, it has been introduced in the EUS evaluation, allowing a quantitative, other than qualitative, analysis of tissue stiffness of the GI tract lesions. Studies have shown high sensitivity and specificity for qualitative elastography, ranging from 92 to 100%, and from 80 to 85%, respectively [40,42], with an overall accuracy of 94% [40]. Quantitative elastography between benign and malignant lesions can yield its sensitivity and specificity up to 100% and 93%, respectively [41].

However, elastography has some intrinsic limitations. These include difficulty of controlling tissue compression by the transducer and targeting the region of interest, excluding nearby structures, presence of motion artifacts (due to respiration and heart beat), and subjective interpretation, though recent studies showed sufficient interobserver agreement (kappa 0.72–0.77) [40,42].

These data suggest that elastography cannot replace EUS-FNA, but it should be considered as a complementary method after a negative EUS-FNA, guiding the tissue sampling for a better biopsy [41], distinguishing non necrotic part of a lesion as well as choosing the more suspicious lymph node [41,42].

## 12. Intraductal Ultrasound (IDUS)

IDUS is a relatively new ultrasonographic modality that uses small-caliber, high-frequency catheters (5–10 Fr, 12–30 MHz). IDUS can visualize both ductal systems and intraluminal strictures. The imaging process can be performed during ERCP, offering complementary information to this procedure. IDUS seems to be particularly beneficial in the differential diagnosis between pancreatic neoplasms and chronic pancreatitis if a main pancreatic duct stenosis is present, with very high sensitivity and specificity, reaching 100% and 92% respectively [13,43].

This method is also beneficial when an IPMN is suspected, with a more detailed resolution imaging comparing to traditional endosonography [8,43,44]. This is also true for the diagnosis of pancreatic mass invading the common bile duct (CBD), with a high sensitivity and specificity, (respectively >90% and >80%) [13].

## 13. Computed Tomography (CT)

CT is a widely used and validated tool for the study of pancreas [8,45]. CT is the primary imaging study for evaluation of patients with symptoms that suggest the presence of the disease. CT is an appropriate initial imaging test because it detects tumors in the pancreas and can be used to stage for resectability and to detect liver metastases. Its sensitivity and specificity have increased considerably due to technical improvements. Multidetector row CT allows imaging of larger volumes of pancreatic tissue while acquiring both arterial and venous phases in shorter periods of time with better delineation of the main pancreatic duct and small intraparenchymal masses. Pancreas imaging protocols can vary from institution to institution, but several principles should be observed to guarantee the best yield. Since the pancreas is supplied by splanchnic arteries, its peak enhancement occurs after the peak enhancement of the aorta (arterial phase) and before the peak enhancement of the liver (hepatic phase) [45]. Studies show that during this phase, there is the maximal contrast between tumor and normal parenchyma to assess the vascular involvement of the lesion [45]. Pancreatic adenocarcinoma usually appears as a hypodense mass, due to hypoperfusion, but it can also be isodense to the surrounding normal tissue [8,13,45]. Sometimes it could be detectable just by secondary signs such as deviation of surrounding vessels or pancreatic duct or common bile duct dilation [8].

The sensitivity for detecting pancreatic adenocarcinoma ranges from 89% to 97%, with higher yield for larger lesions [45]. Legmann *et al.* [46] reported that CT can detect 100% of lesions larger than 1.5 cm, but only 67% of smaller tumors [46]. Bronstein *et al.* [47] found a sensitivity of 77% for pancreatic mass <2 cm and a specificity of 100%. These data could have some bias, since they did not include patients with chronic pancreatitis [47]. Several studies have also compared both radial and linear EUS with helical CT, finding EUS equivalent or superior to CT in detection of pancreatic cancer. EUS sensitivity ranged from 97% to 100%, whereas CT sensitivity was from 68% to 91% [13]. Finally, multidetector CT and EUS were compared, confirming the EUS superiority in detecting lesions that were not seen at CT scan [13].

Comparing these two techniques for T & N staging, EUS was reported to have a superior tumor accuracy (67% *vs.* 41%; p < 0.001), but they were equivalent in lymph node staging (44% *vs.* 47%) [13].

## 14. Magnetic Resonance Imaging (MRI)

MRI is becoming a primary diagnostic tool in the study of pancreatic cancer. Due to its high soft tissue contrast and the multiplicities of different data types that can be acquired, it is reported to be more predictable than CT in the diagnosis of pancreatic tumors, especially for small non-contour-deforming lesions and in detecting liver metastasis and vascular invasion [13,48]. Normal pancreatic parenchyma is hyperintense on noncontrast T1-weighted images, due to the presence of aqueous protein in the pancreatic acinar cells, whereas pancreatic cancer appears as a low-signal (even normal) intensity lesion on precontrast and postcontrast T1-weighted fat suppressed images, because of the abundant fibrous stroma and relatively low tumor vascularity [45,48]. Multiple technological improvements significantly ameliorated the diagnositc yield of MRI over the past decade. This is especially apparent when regarding 3D gradient-echo, which has a better signal-to-noise ratio. This allows thinner slices up to 2 mm and more homogeneous fat saturation [10]. In their prospective comparison of MRI with multislice CT, Fusari *et al.* reported similar accuracy, both for tumor detection and for resectability evaluation, with non statistically significant differences between these two techniques (98/98% and 95/90%, respectively) [49].

Magnetic resonance cholangio-pancreatography (MRCP) has replaced ERCP in the evaluation of patients with suspected pancreatic mass, since it can displays both CBD and pancreatic duct, with high accuracy images (90%) [13]. MRCP also plays a relevant role in suspected IPMN, in detecting, staging and follow up tumors, in order to evaluate the best management for these lesions [50-52]. Kim *et al.* [53] assessed the accuracy of T1-weighted 3D gradient echo sequence MRI, in distinguishing pancreatic cancer from chronic pancreatitis, in patients with focal lesions, reporting a sensitivity of 93% and a specificity of 75%, since it can determine a relative well-defined demarcation of malignant lesions [53].

Although the contrast resolution of MRI, especially for soft tissue, is superior to CT, the spatial resolution of MRI is lower, as is the capacity of acquiring high-quality multiplanar images. Therefore, CT should be generally preferred over MRI for staging pancreatic cancer. MRI should be reserved to those patients in whom iodine contrast administration should be avoidable and in whom CT findings are uncertain [10,48]. MRI could also be useful in characterization and staging of cystic pancreatic tumors, because of the internal structure, which is made of septations and mural nodules, has prognostic value, and is easier to visualize on MRI than on CT [10].

## 15. Positron Emission Tomography (PET)

PET has a marginal role in detection and staging of pancreatic cancer, due to poor spatial resolution, whereas it can be relevant in the detection of distant metastases, as in the evaluation of loco regional tumor recurrence [13,45]. A recent retrospective analysis described pre-radiation FDG-PET parameters as a significant tool in the prediction of prognosis, in patient with locally advanced non-resectable pancreatic cancer [54].

The most used PET radiotracer is 18-fluoro-deoxy-glucose (18-FDG), a glucose analog, which is transported intracellularly via glucose transporters, highly expressed in tumoral cells [8,45]. Its sensitivity in detecting pancreatic cancer ranges from 71 to 92%, with a specificity of 64–94% [8]. Lytras *et al.* recently reported a comparable accuracy to CT in pancreatic assessment, without any additional information in patients with equivocal findings [55]. Frolich *et al.* showed an overall specificity of 95% in detecting liver metastases, with a better yield for larger lesions (specificity of 97% for >1 cm masses, whereas 45% specificity for <1 cm ones) [56]. False positive results can be reported in several inflammatory diseases like pancreatitis, or hyperglycemic states [8].

Preliminary studies have shown that a combination of functional information, provided by FDG-PET and anatomic information provided by CT can be relevant in pancreatic cancer imaging. The positive and negative predictive values of PET/CT for the diagnosis of pancreatic mass are 91% and 69% respectively, allowing a change in patient management. Due to its high positive predictive value in the detection of distal metastases, this novel method should be considered before pancreatic resection [57].

## 16. Conclusions

Pancreatic cancer remains a challenging disease to diagnose at an early stage. CT and MRI are most commonly used in USA to diagnose pancreatic cancer. EUS is the best single modality for tissue acquisition and it is complementary to the other imaging modalities as a staging tool.

## Figures and Tables

**Table 1.** Pancreatic cancer staging.

**Primary Tumor (T)**	
TX	Primary tumor cannot be assessed.
T0	No evidence of primary tumor.
Tis	Carcinoma in situ.
T1	Tumor limited to the pancreas, ≤2 cm in greatest dimension.
T2	Tumor limited to the pancreas, >2 cm in greatest dimension.
T3	Tumor extends beyond the pancreas but without involvement of the celiac axis or the superior mesenteric artery.
T4	Tumor involves the celiac axis or the superior mesenteric artery (unresectable primary tumor).
**Regional Lymph Nodes (N)**	
NX	Regional lymph nodes cannot be assessed.
N0	No regional lymph node metastasis.
N1	Regional lymph node metastasis.
**Distant Metastasis (M)**	
M0	No distant metastasis.
M1	Distant metastasis.

**Table 2.** Pancreatic cancer staging groups (Adapted from [9]).

**Stage**	**T**	**N**	**M**
0	Tis	N0	M0
IA	T1	N0	M0
IB	T2	N0	M0
IIA	T3	N0	M0
IIB	T1	N1	M0
	T2	N1	M0
	T3	N1	M0
III	T4	Any N	M0
IV	Any T	Any N	M1
